# Transcriptional Analysis of Prebiotic Uptake and Catabolism by *Lactobacillus acidophilus* NCFM

**DOI:** 10.1371/journal.pone.0044409

**Published:** 2012-09-19

**Authors:** Joakim Mark Andersen, Rodolphe Barrangou, Maher Abou Hachem, Sampo J. Lahtinen, Yong-Jun Goh, Birte Svensson, Todd R. Klaenhammer

**Affiliations:** 1 Enzyme and Protein Chemistry, Department of Systems Biology, Technical University of Denmark, Lyngby, Denmark; 2 Department of Food, Bioprocessing and Nutrition Sciences, North Carolina State University, Raleigh, North Carolina, United States of America; 3 DuPont Nutrition and Health, Madison, Wisconsin, United States of America; 4 DuPont Nutrition and Health, Kantvik, Finland; University of North Carolina at Charlotte, United States of America

## Abstract

The human gastrointestinal tract can be positively modulated by dietary supplementation of probiotic bacteria in combination with prebiotic carbohydrates. Here differential transcriptomics and functional genomics were used to identify genes in *Lactobacillus acidophilus* NCFM involved in the uptake and catabolism of 11 potential prebiotic compounds consisting of α- and β- linked galactosides and glucosides. These oligosaccharides induced genes encoding phosphoenolpyruvate-dependent sugar phosphotransferase systems (PTS), galactoside pentose hexuronide (GPH) permease, and ATP-binding cassette (ABC) transporters. PTS systems were upregulated primarily by di- and tri-saccharides such as cellobiose, isomaltose, isomaltulose, panose and gentiobiose, while ABC transporters were upregulated by raffinose, Polydextrose, and stachyose. A single GPH transporter was induced by lactitol and galactooligosaccharides (GOS). The various transporters were associated with a number of glycoside hydrolases from families 1, 2, 4, 13, 32, 36, 42, and 65, involved in the catabolism of various α- and β-linked glucosides and galactosides. Further subfamily specialization was also observed for different PTS-associated GH1 6-phospho-β-glucosidases implicated in the catabolism of gentiobiose and cellobiose. These findings highlight the broad oligosaccharide metabolic repertoire of *L. acidophilus* NCFM and establish a platform for selection and screening of both probiotic bacteria and prebiotic compounds that may positively influence the gastrointestinal microbiota.

## Introduction

The microbiota of the human gastrointestinal tract (GIT) can dramatically affect the immune system of the host through increased allergy resistance [1] and modulation of diabetes, obesity [2], [3] and autoimmune bowel disorders [4]. The compositional balance and activity of the microbiota can be positively influenced by probiotic microorganisms [5], or shifted by prebiotic supplementation [6]. One effective strategy to promote positive impacts on both commensal and probiotic microbes is GIT modulation with prebiotic substrates [6]–[9].

Prebiotics are complex carbohydrates that are not digested or absorbed by the host, but catabolized by various commensal and health-promoting members of the GIT bacteria and selectively promoting their growth [10]. Currently, a few carbohydrates are widely accepted as prebiotics, specifically GOS (β-galactooligosaccharides), inulin, FOS (fructo-oligosaccharides) and lactulose [11]. *In vivo* studies, however, have shown increases in the populations of probiotic microbes due to stimulation by candidate prebiotic carbohydrate compounds, e.g. panose [12], polydextrose [13] and lactitol [14]. Advances in the genomics of lactobacilli and bifidobacteria have enabled modeling of transport and catabolic pathways for prebiotic utilization [15]. Only a few such proposed models, however, have been experimentally validated [16]–[18], which hampers accurate functional assignment of novel specificities especially for carbohydrate transporters that are largely uncharacterized biochemically. Recent studies have shown transfer of genes enabling prebiotic catabolism in certain pathogenic strains [19] and growth on prebiotic substrates in mono-cultures of some GIT commensal and pathogenic bacteria [20]. These findings emphasize the need to provide functional scientific support for novel prebiotic candidates and to address the molecular basis for selective prebiotic catabolism by probiotic microbes.

The probiotic microbe *Lactobacillus acidophilus* NCFM has been investigated by in-depth functional studies to reveal the molecular mechanisms for important probiotic traits, such as bile acid resistance [21], involvement of lipoteichoic acid in immunomodulation [22], and positive outcomes reported in human intervention studies using *L. acidophilus* NCFM as a probiotic [23]–[24] and when supplemented as a symbiotic [25]. The potential of *L. acidophilus* NCFM to metabolize a diverse number of oligosaccharides is reflected by the large number of predicted glycoside hydrolases encoded by its genome [26], and by functional studies outlining routes for utilization of various oligosaccharides saccharides [16], [27], [28]. Accurate annotation of genes involved in prebiotic utilization is hampered by the paucity of functional studies, especially of transporters and families of glycoside hydrolases that exhibit a multitude of substrate specificities. The scope of this study was to transcriptionally identify and functionally characterize genomic loci encoding catabolic pathways in *L. acidophilus* NCFM essential for the transport and utilization of a range of potential prebiotics spanning hexose families of α- and β- linked glucosides and galactosides.

## Results

### Carbohydrate dependent differentially expressed gene clusters

Gene expression was measured in *L. acidophilus* NCFM harvested in the early exponential phase and stimulated by glucose compared to 11 different oligosaccharides (Table 1), representing different hexoses in varying groups of carbohydrate linkages. These groups contained the α-galactosides consisting of, raffinose and stachyose; the α-glucosides, isomaltose, isomaltulose, panose and polydextrose; the β-galactosides, lactitol and GOS; and the β-glucosides, β-glucan oligomers, cellobiose and gentiobiose. The overall gene expression pattern for growth on each carbohydrate was represented by cluster analysis (published online, Figure S1) where the global gene expression remained essentially unchanged while the most extensive differential gene regulation was observed for specific gene clusters. The results indicate that *L. acidophilus* NCFM adaptation to and utilization of complex carbohydrate metabolism was regulated at the transcriptional level.

**Table 1:** List of carbohydrates used in this study.

Carbohydrate	Structure^1^	Carbohydrate linkage family	DP 2	Manufacturer or supplier	Purity (as given by manufacturer or supplier)
Glucose	Glc*p*	-	1	Sigma	>99%
GOS	[β-d-Gal*p*-(1–4)]_n_-d-Glc*p*	β-galactoside	2–6	Dupont	>94% DP ≥2
Lactitol	β-d-Gal*p*-(1–4)-d-Glc-ol	β-galactoside	2	Dupont	>99%
Cellobiose	β-d-Glc*p*-(1–4)-d-Glc*p*	β-glucoside	2	Fluka AG	>99%
Gentiobiose^3^	β-d-Glc*p*-(1–6)-d-Glc*p*	β-glucoside	2	Sigma	>98%
β-glucan oligomers	[β-d-Glc*p*-(1–4)]_m_-β-d-Glc*p*-(1–3)-β-d-Glc*p*-[β-d-(1–4)-Glc*p*]_o_	β-glucoside	DP ≥2	Biovelop AB (Sweden)	Essentially free of monosaccharides and cellobiose^4^
Raffinose	α-d-Gal*p*-(1–6)-d-Glc*p*-(α1,β2)-d-Fru*f*	α-galactoside	3	Sigma	>99%
Stachyose	[α-D-Gal*p*-(1–6)]_2_-d-Glc*p*-(α1,β2)-d-Fru*f*	α-galactoside	4	Sigma	>98%
Isomaltose	α-d-Glc*p*-(1–6)-d-Glc*p*	α-glucoside	2	Sigma-Aldrich	>98%
Isomaltulose	α-d-Glc*p*-(1–6)-d-Fru*f*	α-glucoside	2	Dupont	>99%
Panose	α-d-Glc*p*(1–6)-α-d-Glc*p*(1–4)-d-Glc*p*	α-glucoside	3	Sigma	>98%
Polydextrose^5^	Primarily mixed α-glucans, reduced ends	α-glucoside	2–30	Dupont	Essentially free of monosaccharides

Footnotes:

1 n = [1]–[5], m = [0–2] and o = [0–3], ‘n’ is based on oligosaccharide product range of transglycosylation for GOS synthesis as previously described [27]. ‘m’ and ‘o’ are predicted ranges from the theoretical β-glucan repeating polymeric structure and the enzyme used for partial hydrolysis of β-glucan.

2 Degree of polymerization.

3 Isomaltose free, in-house HPAEC-PAD analysis.

4 In-house HPAEC-PAD analysis.

5 Polydextrose Litesse® Ultra (Dupont).

**Figure 1:**
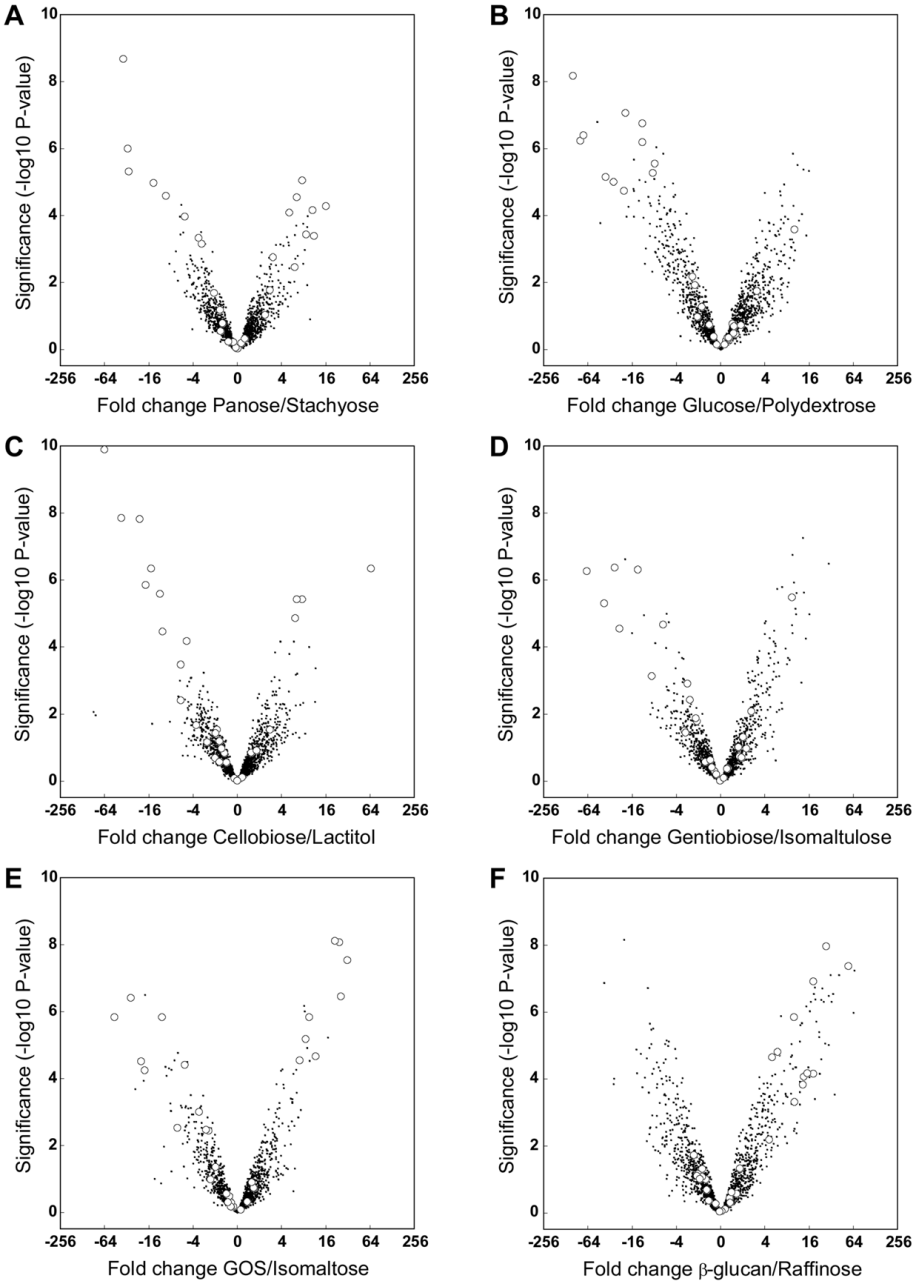
Representative volcano plots of the oligosaccharide-induced differential global transcriptome within *L. acidophilus* NCFM. All genes are shown as black dots (·) and all statistically significant upregulated genes involved with oligosaccharide metabolism (Table 1) are depicted as white circles (○).

Statistical analysis of the global gene expression data was performed by a mixed model ANOVA to identify differentially expressed genes to each oligosaccharide treatment. A range of 1–45 genes were statistically differentially expressed (threshold p = 10^−4,74^ for α = 0.05 using Bonferroni correction) for all treatments. The results of differential gene expression and statistical significance were illustrated by volcano plots that highlighted upregulated genes predicted to be involved in oligosaccharide transport and catabolism (Figure 1) summarized in Table 2 and with a heat map representation of expression of all the identified genes (Figure S2). None of the genes predicted to be involved in oligosaccharide catabolism were upregulated by growth on glucose, consistent with previous findings that glucose is transported by a constitutively expressed phosphoenolpyruvate-dependent sugar phosphotransferase system (PTS) (LBA0452, LBA0455–LBA0457) [27]. Analysis of gene induction patterns by specific oligosaccharides showed a differential expression profile of carbohydrate active proteins (Table 2) depending on the carbohydrate linkages (α- *vs*. β-glycosidic linkages) and the monosaccharide constituents of glucoside and galactoside.

**Table 2:** Statistically significant upregulated genes involved in carbohydrate uptake and catabolism.

ORF	Gene cluster identifier^1^	Gene product annotation	Highest inducing oligosaccharide	Inducing linkage type^2^	Volcano plot (Figure 2)	Fold upregulated	−log_10_ (P-value)
227	A	PTS, EIIC	Gentiobiose	β-glc	2E	9.3	5.48
505	F	β-fructosidase (bfrA), EC 3.2.1.26, GH32	Polydextrose	α-glc	2B	7.9	5.55
506	F	ATP-binding protein (msmK)	Polydextrose	α-glc	2B	11.7	6.75
606	B	PTS permease, EIIBC	Polydextrose	α-glc	2B	81.6	6.23
607	B	Transcriptional regulator, RpiR family	Polydextrose	α-glc	2B	36.9	5.14
608	B	Putative transporter accessory protein	Polydextrose	α-glc	2B	103.3	8.18
609	B	PTS, EIIA	Polydextrose	α-glc	2B	19.9	7.06
724	C	Transcriptional regulator, LicT family	Cellobiose	β-glc	2C	6.1	4.86
725	C	PTS, EIIC	Cellobiose	β-glc	2C	66.0	6.33
876	D	PTS, EIIB	β-glucan oligomers	β-glc	2F	27.1	7.97
877	D	PTS, EIIA	Cellobiose	β-glc	2C	7.6	5.42
884	E	PTS, EIIC	Cellobiose	β-glc	2C	6.4	5.42
1438	G	α-galactosidase (*melA*), EC 3.2.1.22, GH36	Stachyose	α-gal	2E	30.1	5.31
1439	G	ABC, ATP-binding protein (msmK_II_)	Stachyose	α-gal	2E	31.2	5.99
1441	G	ABC, transmembrane permease (msmF_II_)	Stachyose	α-gal	2E	9.3	4.58
1442	G	ABC, substrate-binding protein (msmE_II_)	Stachyose	α-gal	2E	35.9	8.67
1460	H	Putative mucus binding protein (mucBP)	Lactitol	β-gal	2C	11.4	5.59
1461	H	Transcriptional regulator, TetR family	GOS	β-gal	2B	25.5	6.46
1462	H	β-galactosidase (lacA), EC 3.2.1.23, GH42	Lactitol	β-gal	2C	64.0	9.89
1463	H	Lactose permease (lacS)	Lactitol	β-gal	2C	38.2	7.84
1467	H	β-galactosidase large subunit (lacL), EC 3.2.1.23, GH2	GOS	β-gal	2B	24.6	8.06
1684	NA	PTS, EIIA	Polydextrose	α-glc	2B	11.7	6.19
1689	NA	Maltose-6-P glucosidase (malH), EC 3.2.1.122, GH4	Isomaltulose	α-glc	2D	65.9	6.26
1870	NA	Maltose phosphorylase (malP), EC 2.4.1.8, GH65	Polydextrose	α-glc	2B	28.8	5.01

The genes are listed by ascending locus tag numbers. Only the oligosaccharide that elicited the highest induction level is listed for genes that are upregulated by more than one oligosaccharide.

1 Genes not assigned a gene cluster (Figure 3) are listed as not assigned (NA).

2 The predominant glycosidic linkage types have been abbreviated as: α-galactosides (α-gal), α-glucosides (α-glc), β-galactosides (β-gal) and β-glucosides (β-glc).

### β-galactoside differentially induced genes

In the presence of GOS and lactitol, several genes (Table 2) were upregulated (11.4–64 fold) within the locus LBA1460–LBA1468 encompassing genes encoding a galactose-pentose-hexuronide (GPH) LacS permease and two β-galactosidases (GH2 and GH42; CAZy glycoside hydrolase family (GH) classification [29]) together with the Leloir pathway genes (LBA1457–LBA1459 and LBA1469) for galactoside metabolism. These data indicate how these oligosaccharides are transported by the LacS permease and hydrolyzed by the action of two different β-galactosidases into galactose, glucose in the case of GOS and galactose and glucitol for lactitol, which are shunted into the Leloir and glycolytic pathways, respectively, as reported previously [18], [28].

### β-glucoside differentially induced genes

Cellobiose induced genes within two loci (LBA0724–LBA0726 and LBA0877–LBA0884; 6.1–65.8 fold upregulation), both encoding a PTS permease EIIABC and a putative GH1 6-phospho-β-glucosidase. Growth on gentiobiose as a carbon source upregulated (9.2 fold) the PTS permease EIIC (LBA0227), albeit at a lower level by panose (5.4 fold), indicating either a dual specificity of the PTS permease or a more complex transcriptional co-regulation of the transport system. The oligomers obtained by hydrolysis of mixed linkage β-1,3/β-1,4 β-glucan stimulated upregulation of both the cellobiose-induced PTS permease gene cluster mentioned above, and notably the α-glucoside induced gene cluster LBA0606–LBA0609 and LBA1684 (encoding a PTS EIIA component). The patterns of upregulated gene clusters for β-glucosides indicate differential recognition of the β-1,4 and β-1,6 linkages and the specialization of different PTS permeases and their corresponding GH1 enzymes that recognize phosphorylated β-glucosides at the C6 position.

### α-glucoside differentially induced genes

Both isomaltose and isomaltulose upregulated the LBA0606–LBA0609 locus (13.4–65.8 fold), putatively encoding a PTS permease (EIIABC). This regulatory RpiR family protein and a hypothetical protein, together with LBA1684 (11.7 fold upregulated), annotated as a PTS IIA regulatory components. LBA1689 (65.9 fold upregulated) annotated as a GH4 maltose-6-phosphate glucosidase. This suggested that the two α-1,6 linked glucosides are phosphorylated concomitant with their transport by the PTS EIIABC (LBA0606 and LBA0609) permease, and that these phosphorylated disaccharides are hydrolyzed by a specific intracellular (predicted by SignalP [30]) GH4 disaccharide 6-phospho-α-glucosidase into glucose-6-phosphate and either glucose from isomaltose or fructose from isomaltulose, which enter glycolysis. Notably, the trisaccharide panose elicited a similar upregulation pattern as isomaltose, including upregulation of LBA0606–LBA0609 and LBA1689, and also LBA0227. The locus LBA0224–LBA0228 was annotated to include a cellobiose-specific PTS permease EIIC domain, a regulatory protein, and a GH1 6-phospho-β-glucosidase.

The diverse structural elements present in polydextrose constitute a complex oligosaccharide mixture of mostly different α-linked glucosides. Accordingly, a complex upregulation pattern was observed that involved genes encoding both an ABC (LBA500–0504) and a PTS permease (LBA0606) and several hydrolases (LBA0505, LBA1689 and LBA1870). The highest upregulation involved the above PTS permease (LBA0606–0609) together with LBA1870 encoding a GH65 maltose phosphorylase [31] and LBA0505–0506 identified as a part of a locus (LBA0500–LBA0507) determined previously as a FOS metabolism operon [16].

### α-galactoside differentially induced genes

The tetrasaccharide stachyose induced the gene locus LBA1438–LBA1442 (9.3–35.9 fold upregulated) encoding an ABC transporter, a GH36 α-galactosidase and a part of the Leloir pathway enzymes (LBA1458, LBA1459 and LBA1469). This suggests that stachyose is transported into the cytoplasm by this ABC transporter and initially hydrolyzed into galactose and raffinose, which is further processed to galactose and sucrose that subsequently can be phosphorolyzed by LBA1437 encoding a sucrose phosphorylase (GH13_18). This gene cluster was previously found to be upregulated by raffinose [27]. From the DNA microarray presented in the present study, no upregulated genes were involved with oligosaccharide metabolism by stimulation of raffinose, suggesting glucose as an impurity in the medium or raffinose preparation.

### Functional characterization of genes involved with α-galactoside metabolism

**Figure 2:**
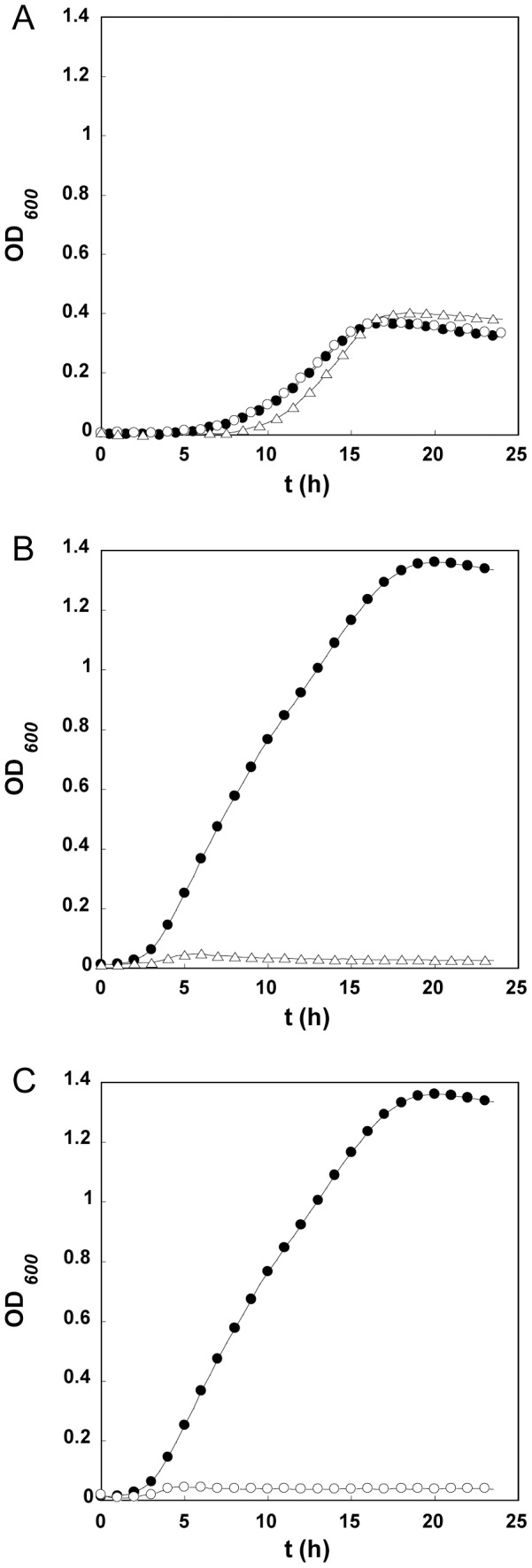
Phenotypic characterization of single gene deletions within *L. acidophilus* NCFM. Growth profiles are shown on galactose (A) and raffinose (B and C) for the mutants within the stachyose-induced gene cluster lacking the GH36 α-galactosidase ΔLBA1438 (Δ) or the solute binding protein component of the ABC transporter ΔLBA1442 (○) compared to *upp*-wildtype (•).

To corroborate the identification of gene clusters from *L. acidophilus* NCFM [25], [27], [32] involved in the metabolism of α-galactosides of the raffinose family oligosaccharides, two single gene deletions were constructed within the stachyose induced locus, i.e. ΔLBA1438 (α-galactosidase) and ΔLBA1442 (solute binding protein of the ABC transporter) using the *upp*-based counterselective gene replacement system [33]. It was predicted by genome mining that *L. acidophilus* NCFM encoded single locus responsible for the transport and hydrolysis of α-galactosides. Phenotypic confirmation of the roles of these genes was accomplished by constructing mutations in these genes. Mutations of LBA1438 and LBA1442 were in-frame deletions of 92% and 91% of the coding regions, respectively. The α-galactosidase (LBA1438) deletion mutant lost the ability to grow on raffinose (Figure 2B), melibiose (α-d-Gal*p*-(1–6)-d-Glc*p*) and stachyose (data not shown). The ability of the LBA1442 mutant to grow on galactose (Figure 2A), but not raffinose (Figure 2C) provides evidence for the specificity of the transporter for α-galactoside oligosaccharides. The phenotypes of single gene deletion variants confirm that the genes identified through differential transcriptomics are functionally crucial for growth on these prebiotic compounds.

### Structure, divergence and function of induced gene clusters

**Figure 3:**
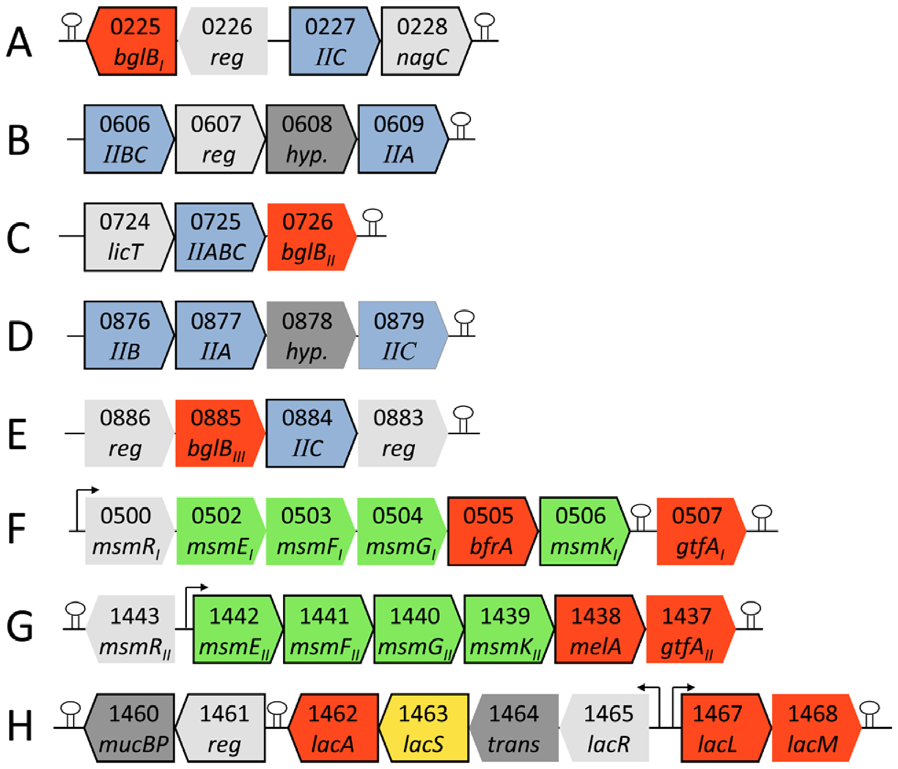
Organization of gene clusters encoding upregulated genes by potential prebiotic oligosaccharide stimulation. All genes are listed with locus tag number and gene name (PTS permeases are shown with domain name; regulators, hypothetical proteins and transposons are abbreviated as reg, hyp. and trans respectively). Gene product functions are colored red for glycoside hydrolases, light grey for transcriptional regulators, blue for PTS permease domains, dark grey for proteins unrelated to carbohydrate metabolism, green for ABC transporter domains and yellow for the GPH permease. All upregulated genes (Table 2) are shown with framed boxes, CRE regulatory sites are represented by arrows and predicted rho-independent transcription terminators [49] by stem loops.

The transcriptional gene induction patterns and the essential roles of single proteins responsible for carbohydrate uptake and catabolism demonstrated how specific gene clusters conferred the ability to utilize the prebiotics investigated in this study. Identification of gene clusters selectively upregulated in response to prebiotic substrates (Figure 3) showed that multiple genes within these operons are typically expressed as single transcripts. However, genes LBA1684 (PTS EIIA component) and LBA1689 (putative maltose-6-phosphate-hydrolase), were predicted *in silico* to be monocistronically transcribed. All gene clusters induced by prebiotic substrates were analyzed for regulatory elements. Catabolite repression elements (CRE) were found upstream of all non-PTS permease containing transcripts and LBA1684, encoding a PTS EIIA. The molecular responses to oligosaccharide stimulation are likely mediated through CRE sites via catabolite control protein A (*ccpA*, LBA0431), phosphocarrier protein HPR (*ptsH*, LBA0639), and HPr kinase/phosphorylase (*ptsK* LBA0676) linking the regulation to the phosphorylation cascade of EI through EIIA to the PTS permeases [27].

**Figure 4:**
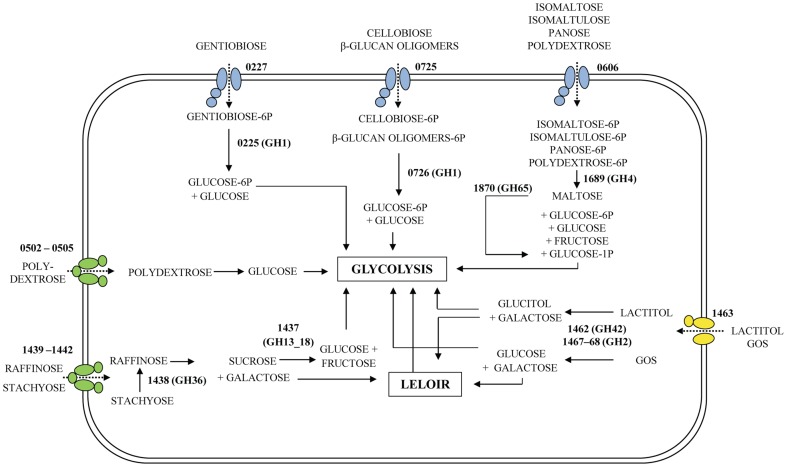
Reconstructed uptake and catabolic pathways in *L. acidophilus* NCFM. Proteins are listed by locus tag LBA numbers, transporters are colored by class (Figure 3) and glycoside hydrolases are listed with GH family number. The polydextrose fraction transported by the ABC transporter (LBA0502–LBA0505) is uncertain and thus the hydrolytic pathway is marked as unknown. The present data outlines the PTS permease LBA0606 (higher level of induction compared to LBA0502–LBA0505) and associated hydrolytic pathway, as the main route of polydextrose utilization by *L. acidophilus* NCFM.

Amino acid sequence comparisons to previously characterized bacterial PTS EIIC trans-membrane substrate binding-domains (Figure S3), including β-1,4 or β-1,6 glucoside specific PTS permeases, showed a clear segregation of LBA0227 and LBA0725, the latter clustering with a functionally characterized cellobiose PTS permease from *L. gasseri* ATCC 33323 [34], consistent with the observed upregulation of LBA0725 on cellobiose. Notably, the PTS permease EIIC domains LBA0879 and LBA0884 were also upregulated by cellobiose, albeit at a lower level than LBA0725. These two proteins clustered distantly on the phylogenetic tree, indicating functional divergence and a likely preference for structurally-related substrates such as sophorose (β-d-Glc*p*-(1–2)-d-Glc*p*), a candidate prebiotic supporting growth of *L. acidophilus* NCFM [12]. A schematic overview (Figure 4) summarizes the uptake and catabolism pathways of potential prebiotic oligosaccharides in *L. acidophilus* NCFM.

Notably, the most highly induced gene in the present study was LBA0608 (Figure S4) encoded a hypothetical protein within a PTS permease locus. No function could be assigned for the protein, which was predicted to have a four transmembrane helical topology using the Phobius prediction tool [35]. The same topology was found for LBA0878 (Figure 3D), another hypothetical protein encoded in locus with a PTS permease, but no significant amino acid sequence similarity was found for the two proteins.

## Discussion

Carbohydrates supplemented for enrichment of specific commensal or probiotic microbes of the GIT can exert selective increases in certain beneficial populations, and decrease pathogens and symptoms of some GIT disorders. Recent studies of prebiotic catabolism [17], [34], [36] have shown a wide array of metabolic capabilities that cannot be deduced based on *in silico* gene annotations or even on experimental work in homologous organisms. The pathways and the molecular elements for transport and catabolism of FOS, lactitol and GOS have been analyzed in *L. acidophilus* NCFM [16], [18], [28]. Herein, mono cultures were used to identify the molecular and genetic foundation for utilization of potential prebiotic compounds, both *in vitro* and/or *in vivo*. This information provides a methodological platform for screening and evaluating potential prebiotic compounds in vivo [37]–[39].

### Importance of carbohydrate transporter variety

The general structure of the identified gene clusters indicates that typically, a three component system consisting of a regulator, transporter and glycoside hydrolase(s) can be sufficient for utilization of potential prebiotics, irrespective of the type of transporter identified (ABC, GPH, or PTS permease, Figure 3). Remarkably, PTS permeases had higher selectivity towards disaccharides, whereas ABC and GPH permeases appeared to be also induced by the longer oligosaccharides e.g. stachyose, and GOS. Furthermore, similar upregulation patterns of gene expression by widely different prebiotics was surprising, notably the FOS-ABC transporter that was also induced by the mixed linkage polydextrose. This suggests that transporters either possess more than one specificity or less stringent molecular recognition of substrates, indicating that a wide range of carbohydrates can be metabolized by *L. acidophilus* NCFM, and likely similar commensal and probiotic bacteria. This capability is also expanded by transporters that possessbroad specificity for oligosaccharides sharing structural elements e.g. the α-1,2 glycosidic linkages found in both FOS and polydextrose.

### Gene deletions confirm GOS and α-galactosides utilization

Functional corroboration of the specificity of prebiotic transport loci has been facilitated by their identification using differential transcriptomics. We previously confirmed that the GPH-type LacS permease is involved in uptake of β-galactosides, GOS and lactitol [28]. Two associated β-galactosidases were involved (LBA1462, GH42, and LBA1467–68, GH2, Figure 3H). The differential expression levels (Figure 1 and Table 2), suggested that GH42 is the main hydrolase for GOS degradation in *L. acidophilus* NCFM. Gene deletions validated both uptake and catabolism for the α-galactosides raffinose, stachyose and melibiose by the locus containing the ABC transport system and GH36 α-galactosidase (LBA1437–LBA1442, Figure 3G).

### Distinct PTS systems and GH1 hydrolases mediate utilization of cellobiose and gentiobiose

Transcriptomics data suggested that the two β-glucoside disaccharide regio-isomers, cellobiose and gentiobiose, only differing in the glucosidic linkage, are internalized by two different PTS systems and hydrolyzed by two different GH1 putative 6-phospho-β-glucosidases having 49% overall sequence identity (Figure 3A and 3C). To validate these findings, the sequences of the PTS transporters and the GH1 hydrolases were analyzed *in silico*. The phylogenetic tree constructed for the PTS systems showed clear segregation of the cellobiose and the gentiobiose induced PTS systems (Figure S3). Notably, the cellobiose induced PTS system clustered together with the functionally characterized cellobiose PTS transporter from *L. gasseri* 33323 that apparently lacks a homolog of the gentiobiose-induced PTS system. Currently there is no biochemical characterization of PTS systems with gentiobiose specificity. Similarly, the two GH1 6-phospho-β-glucosidases from *L. acidophilus* clustered in two distinct GH1 subgroups, whereas a third subgroup was represented by a biochemically and a structurally characterized cellobiose specific GH1 β-1,4-glucosidase [40] (Figure S5). The structure of the 6-phospho-β-glucosidase from *L. plantarum* (PDB: 3QOM, The Midwest Center for Structural Genomics), containing a phosphate ion bound in the active site, has the three conserved residues involved in the recognition of the phosphate moiety of phosphorylated disaccharide substrates (Figure S6A). This, together with sequence alignments (Figure S6A) suggests that the catalytic residues and the phosphate recognition pocket are conserved in LBA0225 and LBA0726, together with all amino acid residues defining the pivotal substrate binding subsite −1, where the non-reducing end 6-phospho-glucosyl residue is bound, are completely conserved (Figure S6B). This is consistent with both putative enzymes being catalytically competent and with their induction together with different PTS transporters, congruent with their recognition of non-reducing end phosphorylated substrates at the C-6 position. Clear differences, however, were observed in amino acid residues of LBA0225 and LBA0726 corresponding to those flanking the putative subsite +1 in the structure of the *L. plantarum* putative 6-phospho-β-glucosidase (Figure S6C), in accordance with the specificity differences suggested by the transcriptomics data. A combination of the GH1 structure-function relationship (Figure S5 and S6) and phylogenetic analysis of PTS permeases (Figure S3) corroborates the transcriptomics findings implicating two β-glucoside isomers in the differential upregulation of the two loci (LBA0225–0228 and LBA0724–0726, Figure 3A and 3C). Improved annotations of both PTS permeases [34] and GH1 6-phospho-glycosidases have previously been limited due to difficulties in working with the transmembrane PTS permeases and the lack of phosphorylated substrates for GH1 or GH4 enzymes. In this light, both transcriptomics and site-specific gene deletions will serve as powerful tools for further functional characterization. The present data offer an important view of the metabolic diversity for *L. acidophilus* NCFM that is differentiated by the type of transporters, GH families and sub-specificities within single GH families.

Remarkably, the LBA0606–0609 locus encoding a PTS permease, was induced by isomaltose and panose revealing a novel pathway for the transport and hydrolysis of short isomaltooligosaccharides, emerging as potential prebiotics [41]. *L. acidophilus* NCFM additionally encodes a canonical GH13 subfamily 31 (GH13_31) glucan-α-1,6-glucosidase homolog to an enzyme from *Streptococcus mutans* shown to be more active on isomaltooligosaccharides longer than isomaltose [42]. However, this locus (LBA0264, GH13_31) was not significantly upregulated in the current study. It is possible that this latter enzyme is induced on longer isomaltooligosaccharides, which may be transported via a different route. Such size dependent differentiation of the utilization pathway has been reported for maltooligosaccharides in other Gram positive bacteria [43]. Furthermore, the locus contained a putative protein with no predictable function (LBA0608), which was the highest induced gene of the study. Sequence analysis indicated a transmembrane topology potentially linking this gene product to the function of the PTS transporter encoded in the locus.

### Comparative genomics of niche specific genes relating to prebiotic utilization

A previous comparative genomics approach predicted LBA1689 orthologs to be selectively found only in GIT associated lactobacilli [44]. This would indicate that the identified novel isomaltose catabolism pathway utilizing the novel isomaltose-6-phosphate hydrolase LBA1689 to be a potential target for α-1,6-glucoside probiotics (e.g. panose and polydextrose) and complementing the conventional route of degradation mediated by the putative α-1,6 glucosidase (LBA0264) encoded in the genome of *L. acidophilus* NCFM.

The important potential for GIT adaption of *L. acidophilus* NCFM by genetic loci encoding specific oligosaccharide utilization is further emphasized from genomic comparisons to the phylogenetically related, but milk adapted *L. helveticus* DPC 4571 [45], where loci, identified in the current study, have been lost through evolution and adaption to milk fermentation for the following oligosaccharides: gentiobiose, FOS, raffinose, isomaltose and panose. These observations underscore how prebiotic stimulation can be considered as a species-specific attribute reflecting evolutionary adaptation to nutritionally rich environments, like the GIT, by either gene gain and functional diversification, or gene-loss associated genome simplification [15].

In conclusion, genes involved in the uptake and catabolism of prebiotic compounds by *L. acidophilus* NCFM were identified using differential transcriptomics. This study revealed the extensive ability of *L. acidophilus* NCFM to utilize a diversity of prebiotic compounds, employing a broad range of carbohydrate uptake systems, including ABC, GPH and PTS transporters, as well as an expansive repertoire of hydrolases that can readily catabolize α- and β-linked glucosides and galactosides.

## Materials and Methods

### *L. acidophilus* NCFM mRNA sample preparation and DNA microarray platform

Whole genome oligonucleotide microarrays were designed as described by Goh et al. [33] with four replicate spots for each of the 1,823 predicted genes. Hybridization quality was assessed as described previously [28]. For preparation of cultures for the DNA microarray transcriptome analysis, a semi-synthetic medium (SSM, [16]) used for cultivation of *L. acidophilus* NCFM was filtered through a 0.22 µm filter and oxygen was removed by the Hungate method [46]. *L. acidophilus* NCFM cultures were propagated in parallel in SSM media supplemented with 1% (w/v) of various carbohydrates as listed for structure and manufacturer in Table 1. Cultures were transferred for five passages on each carbohydrate before harvested at the early logarithmic phase (OD_600_ = 0.35–0.5) by pelleting at 4°C (3,000×*g*, 15 min) and flash freezing the pellets for storage at −80°C.

Cells were mechanically disrupted by beadbeating and total RNA isolated using Trizol-chloroform extraction (Invitrogen, Carlsbad, CA). Genomic DNA was removed with Turbo DNAse (Ambion, Austin, TX), followed by RNA purification using a RNeasy Mini Kit (Qiagen Inc., Valencia, CA) [33].

Reverse transcription of total RNA, fluorescent labeling of cDNA and hybridizations were performed using 20 µg of total RNA for each replicate as described by Goh et al. [33]. Total RNA from each carbohydrate treatment was labeled with both Cyanine3 and Cyanine5 for two technical dye-swapped replicates to each growth condition, and pairwise hybridized using a loop-design for a total of 12 hybridizations.

Hybridized chips were scanned at 10 µm resolution per pixel using a ScanArray Express microarray scanner (Packard BioScience, Meriden, CT) for 16-bit spot intensity quantification. Fluorescent intensities were quantified and background-subtracted using the QuantArray 3.0 software package (Packard Bioscience). Median values were calculated for all ORFs (Open Reading Frames) using tetraplicate intensities and log_2_-transformed before imported into SAS JMP Genomics 4.0 (SAS Institute Inc, Cary, NC) for data analysis. The full data set was interquantile normalized and modeled using a mixed model ANOVA for analysis of the differential gene expression pattern, and visualization using heat maps and volcano plots.

### Bacterial strains and growth conditions

All bacterial strains and plasmids used throughout this study are listed in Table 3. *Lactobacillus* broth cultures were cultivated in MRS (Difco Laboratories Inc., Detroit, MI) or semi-defined medium (SDM) [47], supplemented with 0.5% (w/v) glucose (Sigma-Aldrich, St. Louis, MO)) or 1% (w/v) sucrose (Sigma), galactose (Sigma), melibiose, raffinose (BDH chemicals, Poole, England) and stachyose (Sigma) as carbon sources, in non-shaking batch cultures, aerobically at 37°C or 42°C. Chloramphenicol (Cm, 5 µg/ml) or/and erythromycin (Em, 2 µg/ml) were used when necessary for selection. *Escherichia coli* strains were cultivated in Brain Heart Infusion medium (Difco) aerobically at 37°C with aeration, and Em (150 µg/ml) and/or kanamycin (Km, 40 µl/ml) were/was added for selection. Solid media were prepared by the addition of 1.5% (w/v) agar (Difco).

**Table 3:** Strains and plasmids used in the study.

Strain or plasmid	Characteristics	Reference or source
*E. coli* strains		
NCK1831	EC101: RepA^+^ JM101; Km^r^; *repA* from pWV01 integrated in chromosome; host for pORI-based plasmids	[54]
NCK1911	NCK1831 harboring pTRK935	[33]
NCK2122	NCK1831 harboring pTRK1013	This study
NCK2124	NCK1831 harboring pTRK1014	This study
*L. acidophilus* strains		
NCFM	Human intestinal isolate	[26]
NCK1909	NCFM carrying a 315 bp in-frame deletion in the *upp* gene	[33]
NCK1910	NCK1909 harboring pTRK669, host for pORI-based counter selective integration vector	[33]
NCK2123	NCK1909 carrying a 2029 bp in-frame deletion in the *melA* gene	This study
NCK2125	NCK1909 carrying a 1141 bp in-frame deletion in the *msmE* gene	This study
Plasmids		
pTRK669	Ori (pWV01], Cm^r^ RepA^+^	[55]
pTRK935	pORI28 derived with an inserted *upp* expression cassette and *lacZ'* from pUC19, serves as counterselective integration vector, Em^r^	[33]
pTRK1013	pTRK935 with a mutated copy of *melA* cloned into BamHI/EcoRI sites	This study
pTRK1014	pTRK935 with a mutated copy of *msmE_II_* cloned into BamHI/EcoRI sites	This study

### Construction and phenotypic determination of deletion mutants in the α-galactoside gene cluster

Genomic DNA of *L. acidophilus* NCFM was isolated by the method of Walker and Klaenhammer [48] or by the Mo Bio Ultraclean microbial DNA isolation kit (Mo Bio Laboratories, Carlsbad, CA). Plasmid DNA from *E. coli* was isolated using a QIAprep Spin miniprep kit (Qiagen). Restriction enzymes (Roche Molecular Biochemicals, Indianapolis, IN) were applied according to the instructions supplied by the manufacturer. DNA ligation was done using T4 DNA ligase (New England Biolabs, Beverly, MA) as directed by the manufacturers' recommendations. All PCR primers were synthesized by Integrated DNA Technologies (Coralville, IA). PCR reactions, preparation and transformation of competent *L. acidophilus* NCFM and *E. coli* cells, analysis by agarose gel electrophoresis, and in gel purification were done as described by Goh et al. [33].

The construction of a *Δupp* isogenic mutant with in-frame DNA excision of the LBA1438 and LBA1442 coding region was done according to Goh et al. [33]. In short, the upstream and downstream flanking regions (approximate length of 750 bp each) of the deletion targets were PCR-amplified either with the 1438A/1438B and 1438C/1438D or 1442A/1442B and 1442C/1442D primer pairs, respectively, and fused by splicing by overlap extension PCR (SOE-PCR). The SOE-PCR products were ligated into pTRK935 linearized with compatible ends (BamHI and EcoRI for all constructs), and transformed into NCK1831. The resulting recombinant plasmids, pTRK1013 and pTRK1014, harbored in NCK2122 and NCK2124, were transformed into NCK1910 harboring pTRK669, for chromosomal integration and following DNA excision to generate the Δ*melA* or Δ*msmE* genotypes respectively. Confirmation of DNA deletion was done by PCR and DNA sequencing using primer pair 1438UP/1438DN and 1442UP/1442DN (see Table S1).

Carbohydrate utilization of the gene deletion mutants was tested by comparative growth to wild type *L. acidophilus* NCFM and NCK1909 (*upp* mutant and parent strain of the Δ*melA* and Δ*msmE_II_* mutants). All strains were grown in SDM supplemented with 1% (w/v) glucose before inoculation (1% (v/v) of an overnight culture into SDM supplemented with 1% (w/v) of the following carbohydrates in separate batches: raffinose, stachyose, sucrose and galactose. Growth was monitored by measuring optical density (OD*_600_*) using a Fluostar spectrophotometer (BMG Labtech, Cary, NC)) in triplicate wells of a 96-well plate (200 µl per well) covered with an airtight seal.

### Microarray Data Submission

All raw data have been deposited in the GEO database under accession GSE35968 and complies with the MIAME guidelines.

## Supporting Information

Figure S1 Hierarchical two-way clustering of the global gene expression patterns across 1823 genes for all carbohydrate growth conditions. Up-regulated genes are shown in red while downregulated genes are shown in blue.
(TIF)

Figure S2 Two-way clustering of identified statistically significant genes involved in carbohydrate utilization listed with locus tag LBA numbers. Up-regulated genes are shown in red while downregulated genes are shown in blue.
(TIF)

Figure S3 Phylogenetic diversity of β-glucoside specific PTS EIIC domains of *L. acidophilus* NCFM. Clustering of identified *L. acidophilus* NCFM PTS EIIC domains (highlighted in bold) is visualized by a phylogenetic tree where representative sequences are used to illustrate functional segregation. Reference sequences are from *L. gasseri* 33323 or PTS EIIC homologs (>50% amino acid identity) from reference genomes of the human microbiome [50]. PTS EIIC domain sequences are identified by homology search of the Swiss-prot database [51] and all phylogenetic distances were calculated using ClustalW2 [52] All phylogenetic trees were visualized using MEGA5 (http://www.megasoftware.net/). All known substrate specificities are given in parentheses, otherwise amino acid identity to LBA0227 is stated. Uniprot references: *Bacillus subtilis*, lichenan (P46317), *Geobacillus stearothermophilus*, cellobiose (Q45400), *Bacillus subtilis*, mannobiose and cellobiose (O05507), *Lactobacillus casei*, lactose (P24400), *Streptococcus mutants*, lactose (P50976), *Lactococcus lactis*, lactose (P23531) and *Escherichia coli*, *N,N*′-diacetylchitobiose (P17334.2).
(TIF)

Figure S4 Gene expression levels for highest induced gene (LAB0608) and gene cluster. The locus encoded an α-1,6-glucoside specific PTS EIIBC (LBA0606), a transcriptional regulator (LBA0607), a putative transporter associated protein (LBA0608) and PTS EIIA component (LBA0609) showed consistent high expression of the full locus indicating a functional connection of LBA0608 and PTS permease uptake. Values are given as the mean value (○) of the technical replicates represented by bars.
(TIF)

Figure S5 Phylogenetic relationship of the two identified 6-phospho-β-glucosidases (LBA0225 and LBA0726) compared to characterized GH1 enzymes (the *Bacillus circulans* subsp. *alkalophilus* β-1,4-glucosidase (gi: 308070788) and *L. plantarum* 6-phospho-β-glucosidase structure (PDB accession: 3QOM)). The closest homologs, all listed by gi-number, specie and strain name, were identified for LBA0227, LBA726 and the cellobiose specific *Bacillus circulans* subsp. *alkalophilus* β-1,4-glucosidase by BLAST searching against the non-redundant database [53] and all phylogenetic distances were calculated using ClustalW2 [52]. All phylogenetic trees were visualized using MEGA5 (http://www.megasoftware.net/). Distinct clustering even of related taxa was observed reflecting differential substrate specificity for cluster (A) proposed to be gentiobiose-6-phosphate specific with LBA0227 highlighted in bold, (B) cellobiose specific as represented by *Bacillus circulans* subsp. *alkalophilus* β-1,4-glucosidase and (C) proposed to be cellobiose-6-phosphate specific with LBA0726 highlighted in bold.
(TIF)

Figure S6 Functionally pivotal residues in 6-phospho-β-glucosidases of GH1. (A) The selected segments of the multi-sequence alignment used to construct the phylogenetic tree (Figure S5 cluster A and C), showing conserved and variable putative substrate interacting residues of LBA0225 and LBA0726. Conserved residues of the −1 subsite are marked with green, the catalytic acid/base (E180) and nucleophile (E375) are marked with purple, the putative +1 subsite is marked with cyan and the residues that recognize the phosphate moiety in the phosphate binding pocket are marked with grey. All numbering corresponds to the *L. plantarum* 6-phospho-β-glucosidase structure (PDB accession: 3QOM) as reference, also used to depict functionally important residues in 6-phospho-β-glucosidases of GH1; (B) highly conserved active site residues are colored as in (A) and shown in sticks. (C) A surface representation of the active site (40% transparency) showing (cyan sticks) the proposed putative subsite +1 specificity determinants distinguishing 6-phospho-β-1,6-glucosides represented by LBA0227, from 6-phospho-β-1,4-glucosides represented by LBA0725. The catalytic residues are surface colored in purple to denote the position of the −1 subsite. Pymol was used for molecular rendering (The PyMOL Molecular Graphics System, Version 1.3, Schrödinger, LLC.)
(TIF)

Table S1 Primers used for construction of gene deletion mutants. Restriction sites are highlighted in bold and underlined.
(DOCX)
