# Supplementary figures and images for: Transcriptional Analysis of Prebiotic Uptake and Catabolism by Lactobacillus acidophilus NCFM
Source: PLoS One. 2012 Sep 19;7(9):e44409. doi: 10.1371/journal.pone.0044409 (PMC3446993; doi:10.1371/journal.pone.0044409)

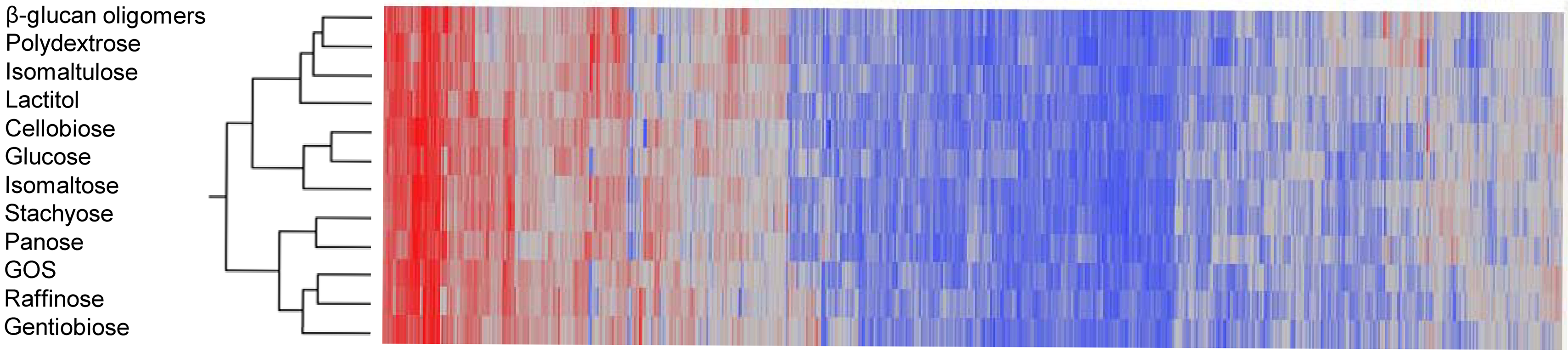

Supplement: Figure S1 — Hierarchical two-way clustering of the global gene expression patterns across 1823 genes for all carbohydrate growth conditions. Up-regulated genes are shown in red while downregulated genes are shown in blue. (TIF) [file pone.0044409.s001.tif]

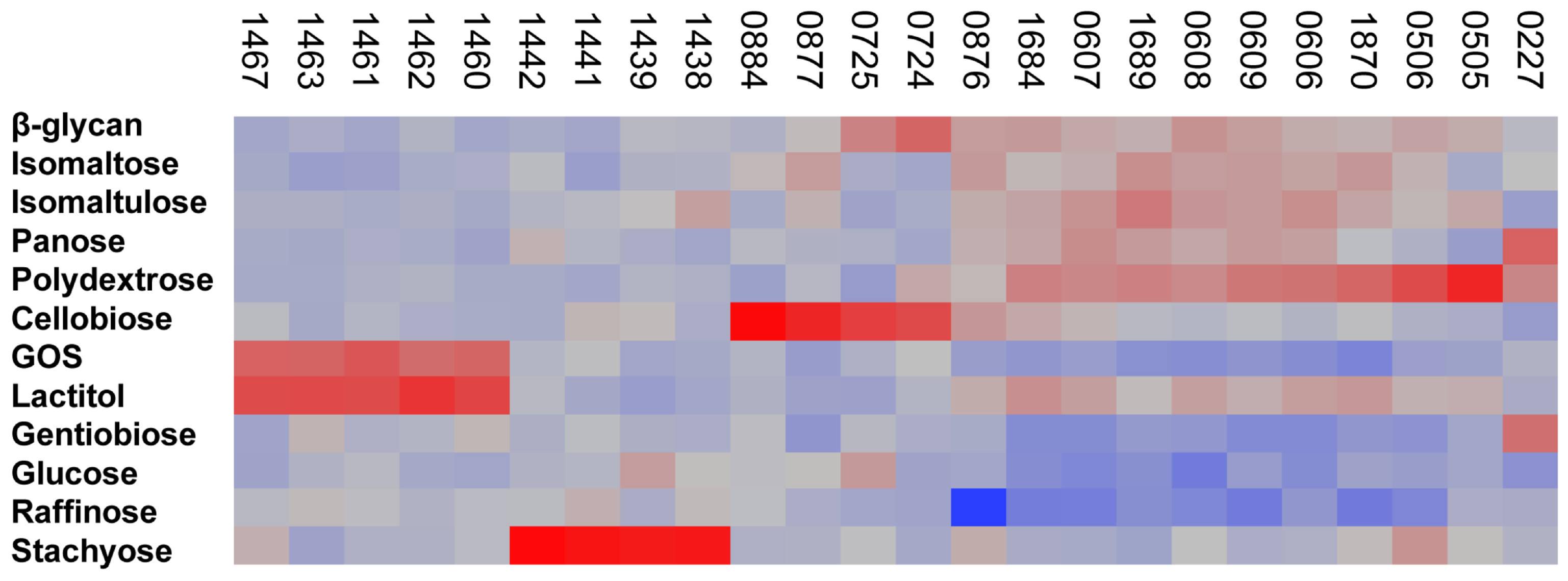

Supplement: Figure S2 — Two-way clustering of identified statistically significant genes involved in carbohydrate utilization listed with locus tag LBA numbers. Up-regulated genes are shown in red while downregulated genes are shown in blue. (TIF) [file pone.0044409.s002.tif]

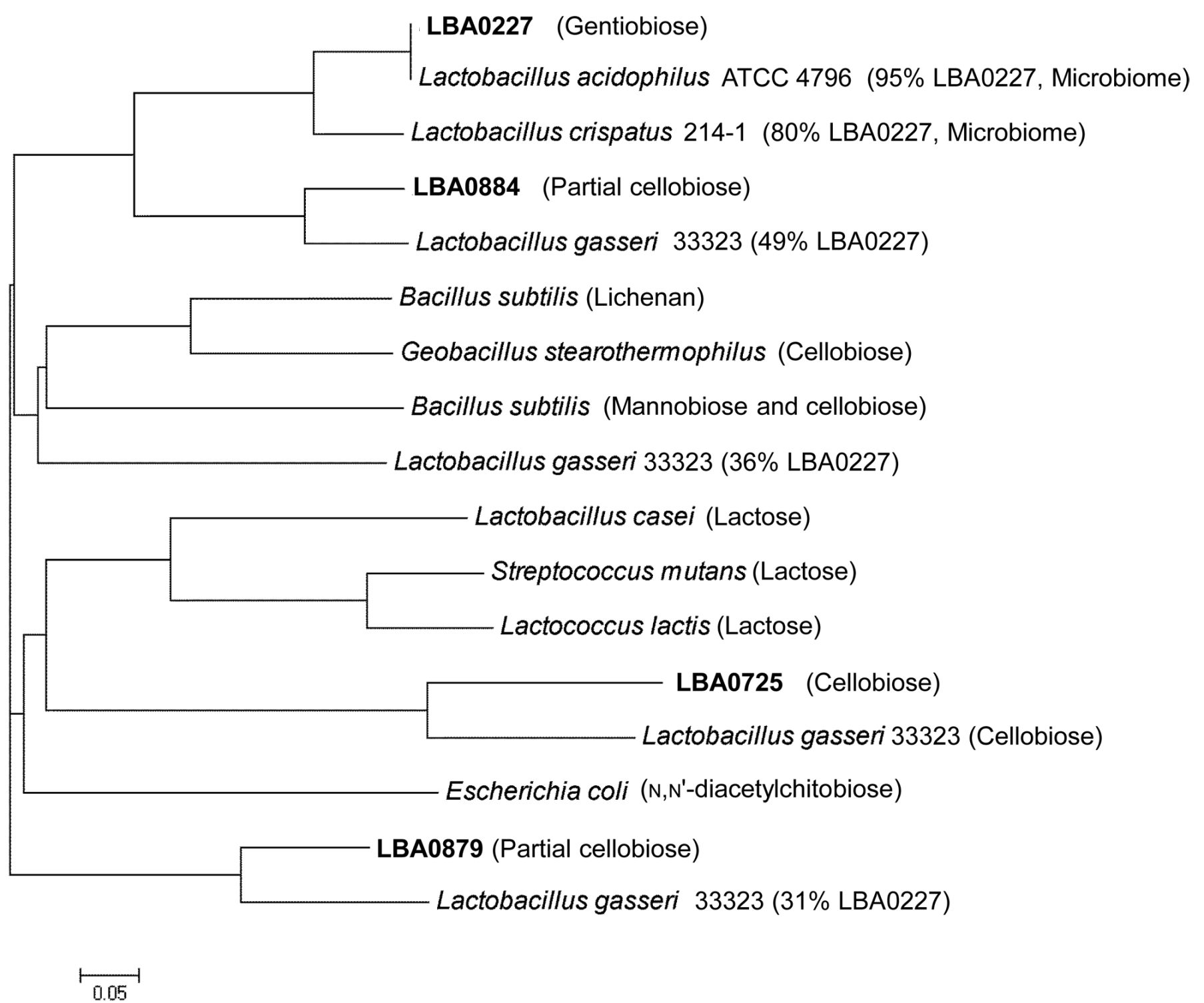

Supplement: Figure S3 — Phylogenetic diversity of β-glucoside specific PTS EIIC domains of L. acidophilus NCFM. Clustering of identified L. acidophilus NCFM PTS EIIC domains (highlighted in bold) is visualized by a phylogenetic tree where representative sequences are used to illustrate functional segregation. Reference sequences are from L. gasseri 33323 or PTS EIIC homologs (>50% amino acid identity) from reference genomes of the human microbiome [50]. PTS EIIC domain sequences are identified by homology search of the Swiss-prot database [51] and all phylogenetic distances were calculated using ClustalW2 [52] All phylogenetic trees were visualized using MEGA5 (http://www.megasoftware.net/). All known substrate specificities are given in parentheses, otherwise amino acid identity to LBA0227 is stated. Uniprot references: Bacillus subtilis, lichenan (P46317), Geobacillus stearothermophilus, cellobiose (Q45400), Bacillus subtilis, mannobiose and cellobiose (O05507), Lactobacillus casei, lactose (P24400), Streptococcus mutants, lactose (P50976), Lactococcus lactis, lactose (P23531) and Escherichia coli, N,N′-diacetylchitobiose (P17334.2). (TIF) [file pone.0044409.s003.tif]

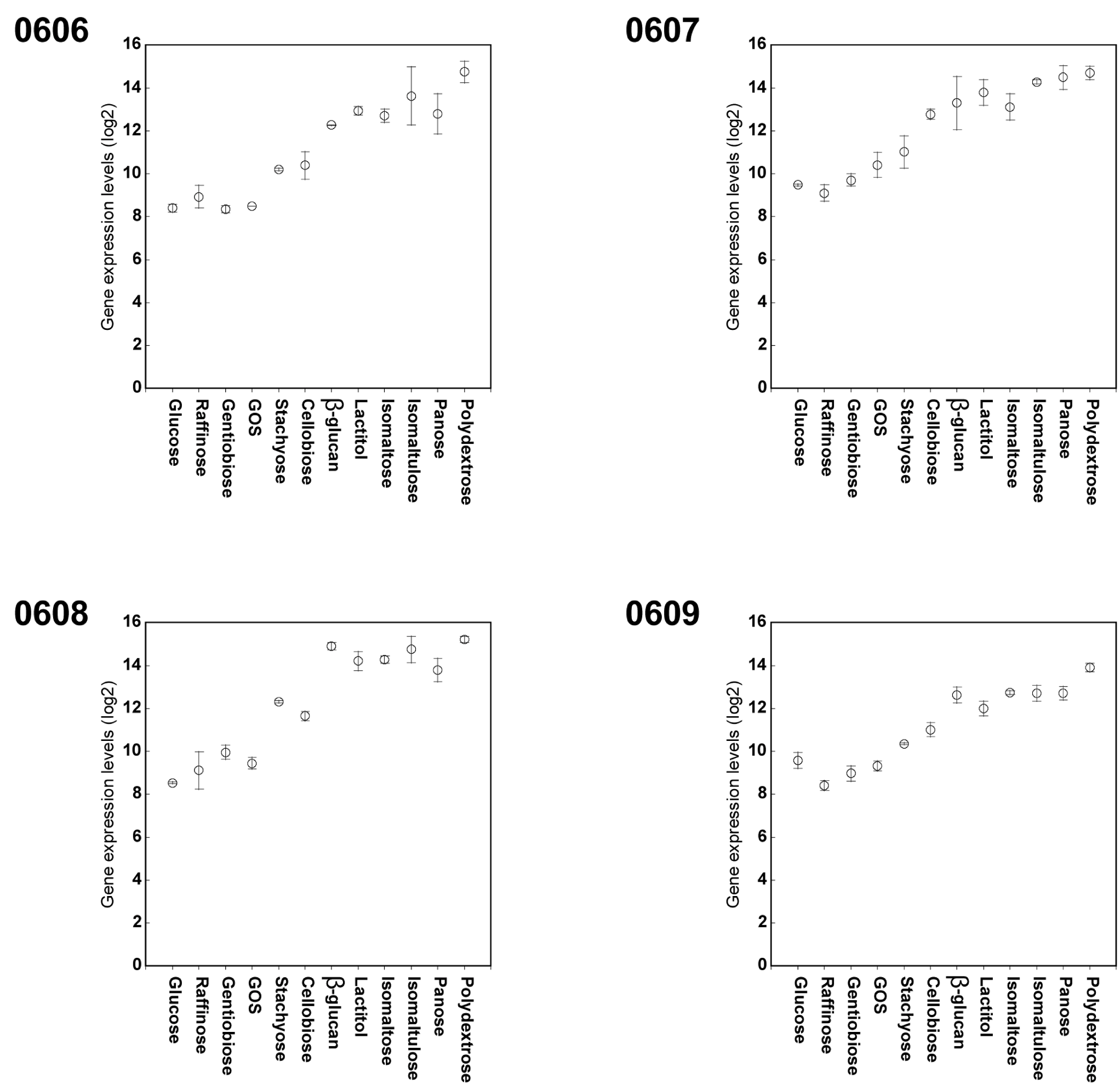

Supplement: Figure S4 — Gene expression levels for highest induced gene (LAB0608) and gene cluster. The locus encoded an α-1,6-glucoside specific PTS EIIBC (LBA0606), a transcriptional regulator (LBA0607), a putative transporter associated protein (LBA0608) and PTS EIIA component (LBA0609) showed consistent high expression of the full locus indicating a functional connection of LBA0608 and PTS permease uptake. Values are given as the mean value (○) of the technical replicates represented by bars. (TIF) [file pone.0044409.s004.tif]

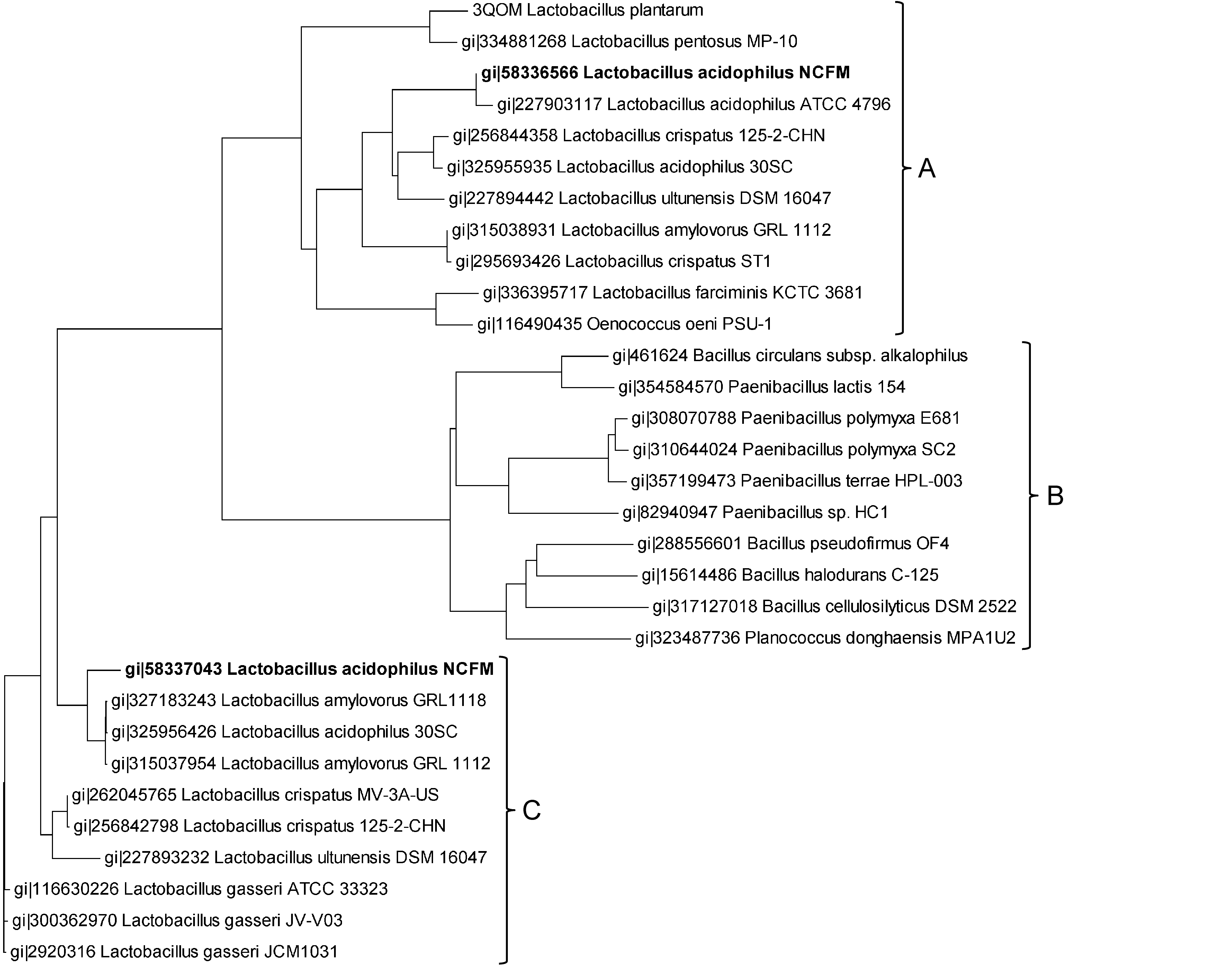

Supplement: Figure S5 — Phylogenetic relationship of the two identified 6-phospho-β-glucosidases (LBA0225 and LBA0726) compared to characterized GH1 enzymes (the Bacillus circulans subsp. alkalophilus β-1,4-glucosidase (gi: 308070788) and L. plantarum 6-phospho-β-glucosidase structure (PDB accession: 3QOM)). The closest homologs, all listed by gi-number, specie and strain name, were identified for LBA0227, LBA726 and the cellobiose specific Bacillus circulans subsp. alkalophilus β-1,4-glucosidase by BLAST searching against the non-redundant database [53] and all phylogenetic distances were calculated using ClustalW2 [52]. All phylogenetic trees were visualized using MEGA5 (http://www.megasoftware.net/). Distinct clustering even of related taxa was observed reflecting differential substrate specificity for cluster (A) proposed to be gentiobiose-6-phosphate specific with LBA0227 highlighted in bold, (B) cellobiose specific as represented by Bacillus circulans subsp. alkalophilus β-1,4-glucosidase and (C) proposed to be cellobiose-6-phosphate specific with LBA0726 highlighted in bold. (TIF) [file pone.0044409.s005.tif]

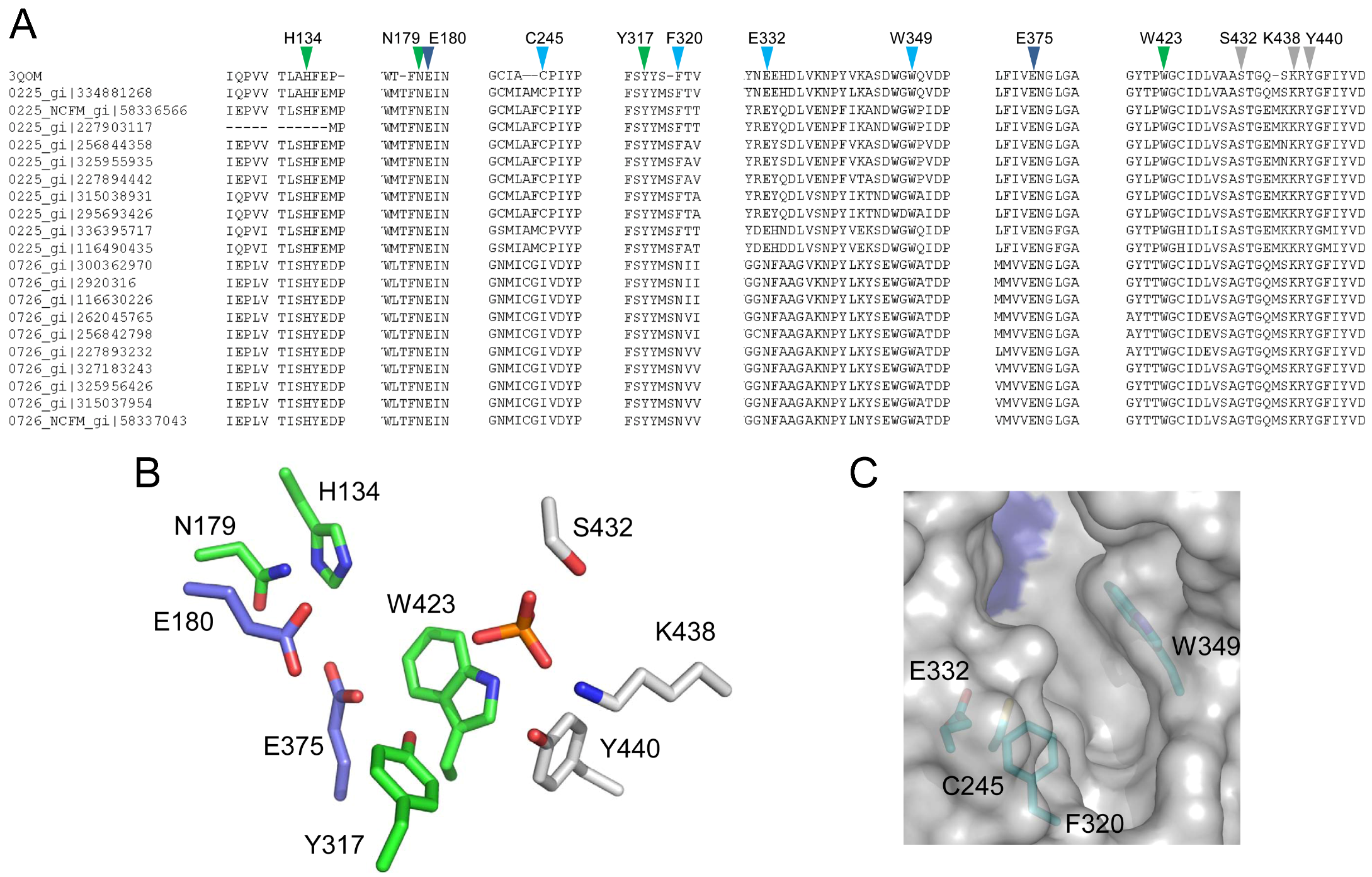

Supplement: Figure S6 — Functionally pivotal residues in 6-phospho-β-glucosidases of GH1. (A) The selected segments of the multi-sequence alignment used to construct the phylogenetic tree (Figure S5 cluster A and C), showing conserved and variable putative substrate interacting residues of LBA0225 and LBA0726. Conserved residues of the −1 subsite are marked with green, the catalytic acid/base (E180) and nucleophile (E375) are marked with purple, the putative +1 subsite is marked with cyan and the residues that recognize the phosphate moiety in the phosphate binding pocket are marked with grey. All numbering corresponds to the L. plantarum 6-phospho-β-glucosidase structure (PDB accession: 3QOM) as reference, also used to depict functionally important residues in 6-phospho-β-glucosidases of GH1; (B) highly conserved active site residues are colored as in (A) and shown in sticks. (C) A surface representation of the active site (40% transparency) showing (cyan sticks) the proposed putative subsite +1 specificity determinants distinguishing 6-phospho-β-1,6-glucosides represented by LBA0227, from 6-phospho-β-1,4-glucosides represented by LBA0725. The catalytic residues are surface colored in purple to denote the position of the −1 subsite. Pymol was used for molecular rendering (The PyMOL Molecular Graphics System, Version 1.3, Schrödinger, LLC.) (TIF) [file pone.0044409.s006.tif]
