# Supplementary material for: Transcriptional Analysis of Prebiotic Uptake and Catabolism by Lactobacillus acidophilus NCFM
Source: PLoS One. 2012 Sep 19;7(9):e44409. doi: 10.1371/journal.pone.0044409 (PMC3446993; doi:10.1371/journal.pone.0044409)
Supplement: Table S1 — Primers used for construction of gene deletion mutants. Restriction sites are highlighted in bold and underlined. (DOCX) [file pone.0044409.s007.docx]

Table S1: Primers used for construction of gene deletion mutants. Restriction sites are highlighted in bold and underlined.

| LBA1438 upstream flanking region | | |
| --- | --- | --- |
|  | 1438A | CGC**GGATCC**cgaaccactatccaaccttga |
|  | 1438B | CCACCATCTTCAATAGAAAGC |
| LBA1438 downstream flanking region | | |
|  | 1438C | GCTTTCTATTGAAGATGGTGGACCTTGGCTTTTATGATCCTATTG |
|  | 14383D | CCG**GAATTC**cccaaatttctggctctacaa |
| LBA1438 DNA excision control | | |
|  | 1438UP | caccaaagtaggcgatactgaa |
|  | 1438DN | acagcccccttcaagtcttc |
| LBA1442 upstream flanking region | | |
|  | 1442A | CGC**GGATCC**TTGATGCAAGTAACGCTGAGA |
|  | 1442B | GTAGCCATCATGACTCCAATTAG |
| LBA1442 downstream flanking region | | |
|  | 1442C | CTAATTGGAGTCATGATGGCTACGGTAATAAACAACAAATGGTTAATG |
|  | 1442D | CCG**GAATTC**GGGAGTTCAATCTTCCAGAAA |
| LBA1442 DNA excision control | | |
|  | 1442UP | aaggccaaatgacaataatgc |
|  | 1442DN | gcaccttgaactaatgggaaa |
